# Breast Cancer in Elderly Caucasian Women—An Institution-Based Study of Correlation between Breast Cancer Prognostic Markers, TNM Stage, and Overall Survival

**DOI:** 10.3390/cancers7030846

**Published:** 2015-07-31

**Authors:** Amila Orucevic, Matthew Curzon, Christina Curzon, Robert E. Heidel, James M. McLoughlin, Timothy Panella, John Bell

**Affiliations:** 1 Department of Pathology, Graduate School of Medicine, University of Tennessee Medical Center at Knoxville, Knoxville, TN 37920, USA; E-Mail: mcurzon@mc.utmck.edu; 2 Department of Family Medicine, Graduate School of Medicine, University of Tennessee Medical Center at Knoxville, Knoxville, TN 37920, USA; E-Mail: ccurzon@mc.utmck.edu; 3 Department of Surgery, Graduate School of Medicine, University of Tennessee Medical Center at Knoxville, Knoxville, TN 37920, USA; E-Mails: rheidel@mc.utmck.edu (R.E.H.); jmcloughlin@mc.utmck.edu (J.M.M.); jlbell@mc.utmck.edu (J.B.); 4 Department of Medicine, Graduate School of Medicine, University of Tennessee Medical Center at Knoxville, Knoxville, TN 37920, USA; E-Mail: tpanella@mc.utmck.edu

**Keywords:** breast cancer prognostic markers, TNM staging, overall survival, Caucasian women

## Abstract

There is still a paucity of data on how breast cancer (BC) biology influences outcomes in elderly patients. We evaluated whether ER/PR/HER2 subtype and TNM stage of invasive BC had a significant impact on overall survival (OS) in a cohort of 232 elderly Caucasian female patients (≥70 year old (y/o)) from our institution over a ten-year interval (January 1998–July 2008). Five ER/PR/HER2 BC subtypes classified per 2011 St. Gallen International Expert Consensus recommendations were further subclassified into three subtypes (traditionally considered “favorable” subtype-ER+/PR+/HER2−, and traditionally considered “unfavorable” BC subtypes: HER2+ and triple negative). OS was measured comparing these categories using Kaplan Meier curves and Cox regression analysis, when controlled for TNM stage. The majority of our patients (178/232 = 76.8%) were of the “favorable” BC subtype; 23.2% patients were with “unfavorable” subtype (HER2+ = 12% (28/232) and triple negative = 11.2% (26/232)). Although a trend for better OS was noted in HER2+ patients (68%) *vs*. 56% in ER+/PR+ HER2− or 58% in triple negative patients, “favorable” BC subtype was not significantly predictive of better OS (*p* = 0.285). TNM stage was predictive of OS (*p* < 0.001). These results are similar to our published studies on Caucasian BC patients of all ages in which ER/PR/HER2 status was not predictive of OS, irrespective of classification system used.

## 1. Introduction

The American Cancer Society estimates that 231,840 women will be diagnosed with breast carcinoma and 40,290 women will die from it in 2015 [1]. While the lifetime risk of developing breast carcinoma is still 1 in 8 women (similar from 2003–2011) [2,3], the risk that a ≥70 y/o woman will be diagnosed with breast cancer during the subsequent 10 years is much higher when compared to women ≤70 y/o. The risk reaches 3.82% or 1 in 26 women for a 70 year old woman, almost twice the risk of a 50 year old woman (2.38% or 1 in 42 women) [2].

In spite of the fact that the incidence of breast carcinoma increases with age, with approximately 30% of new breast carcinoma cases being diagnosed in patients 70 years and older [3], there is still a paucity of data on how breast cancer biology influences outcomes in elderly patients. A few studies demonstrated that breast carcinoma in elderly patients have a higher probability of “favorable” tumor biology, including hormone receptor positive (ER and/or PR positive) and HER2 negative breast carcinomas, as well as node-negative carcinomas [4,5,6]. However, in spite of a higher probability of “favorable” tumor biology, almost 50% of deaths from breast carcinoma occur in the elderly patient population (≥70 y/o) [3]. Some studies have attributed this observation to an under-representation of elderly patients in clinical trials and, consequently, a lack of standardized treatments and clear management guidelines for these patients [6,7]. For example, based on the newest NCCN guidelines, breast cancer edition, “there are limited data to make chemotherapy recommendations for those >70 y/o. Treatment should be individualized with consideration of comorbid conditions” [8]. Currently, individualized, and therefore highly variable treatment approaches, are often recommended for elderly patients rather than the standardized algorithmic approach used for management of patients younger than 70 years of age.

We have previously shown in two of our institution’s studies that ER/PR/HER2 status was not predictive of overall survival of Caucasian female breast carcinoma patients, irrespective of the classification system used, while TNM stage was predictive of overall survival [9,10]. We have now evaluated the same cohort of patients for the prognostic value of old age (≥70 y/o) on overall survival, when controlled for cancer hormonal subtype and pathologic tumor characteristics. Specifically, we assessed whether ER/PR/HER2 subtype and TNM stage of invasive breast carcinoma had significant impact on overall survival in a cohort of 232 elderly Caucasian female patients (≥70 y/o) from our institution at the 10 year interval (January 1998–July 2008), with 1 August 2013 as the last day of follow-up.

## 2. Results

The majority of our patients (178/232 = 76.8%) were of the “favorable” breast carcinoma subtype (ER+ and/or PR+, HER2−), subdivided to the luminal A-like and luminal B/HER2 negative-like subtypes. 23.2% patients were of traditionally considered “unfavorable” subtype: (1) HER2+ subtype = 12% (28/232), subdivided to luminal B/HER2 positive-like subtype (16/232) and HER2 positive/non-luminal like subtype (12/232) and (2) triple negative subtype = 11.2% (26/232) (Table 1).

**Table 1:** Clinicopathologic characteristics of invasive carcinomas.

	Luminal A-like “Favorable” Subtype	Luminal B/HER2− like “Favorable” Subtype	Luminal B/HER2+ like Traditionally “Unfavorable” Subtype	Nonluminal/HER2+ like Traditionally “Unfavorable” Subtype	Triple Negative-like “Unfavorable” Subtype
**ER/PR/HER2 frequency**	104/232 = 44.8%	74/232 = 32%	16/232 = 6.8%	12/232 = 5.2%	26/232 = 11.2%
**Age (mean value)**	78.2	77	74.4	74.9	76.3
**Tumor grade**					
**Grade 1**	*N* = 51	*N* = 13	*N* = 3	*N* = 0	*N* = 1
**Grade 2**	*N* = 51	*N* = 40	*N* = 5	*N* = 1	*N* = 8
**Grade 3**	*N* = 2	*N* = 21	*N* = 7	*N* = 10	*N* = 17
**Tumor size (mm) (mean value)**	19.37	23.97	17.25	24.27	23.5
**TNM stage**					
**Stage I**	*N* = 58	*N* = 29	*N* = 10	*N* = 7	*N* = 12
**Stage II**	*N* = 32	*N* = 33	*N* = 4	*N* = 2	*N* = 7
**Stage III**	*N* = 8	*N* = 10	*N* = 2	*N* = 3	*N* = 5
**Stage IV**	*N* = 6	*N* = 2	*N* = 0	*N* = 0	*N* = 2
**Survival months (mean value)**	72.2	78	101.1	72.9	64.8
**% of alive patients at the end of the study**	55/104 = 53%	44/74 = 59%	11/16 = 69%	8/12 = 67%	15/26 = 58%

(*N*) = number of patients.

The oldest patients were in the luminal A-like group (mean age 78.2), and the youngest were in the luminal B/HER2 positive-like subtype (mean age 74.4) (Table 1). The majority of patients had invasive ductal carcinoma as a histologic tumor type, regardless of ER/PR/HER2 subtype (163/232 = 70%), followed by invasive lobular carcinoma (34/232 = 14.6%). The most common grade of invasive carcinoma was grade 1 and grade 2 for luminal A-like, grade 2 for luminal B/HER2 negative-like, and grade 3 for luminal B/HER2 positive-like, HER2 positive/non-luminal like, and triple negative subtypes (Table 1). The most frequent TNM stage for all subtypes was stage I, with the exception of luminal B/HER2 negative subtype that had stage II as the most frequent stage (Table 1).

The majority of our patients underwent modified radical mastectomy as the final surgical procedure (143/232 = 61.6%). Table 2 shows post-surgery treatments that our patients received: only 32.3% of patients received radiation therapy, 21% received chemotherapy, and just slightly over half of hormone receptor-positive patients received hormonal therapy (57.2%). Only 5 of 28 HER2+ patients were diagnosed with HER2 positive breast carcinoma after October 2005, when anti-HER2 adjuvant therapy became a standard treatment for patients with HER2+ breast carcinoma in our institution. Three of these 5 patients (60%) received anti-HER2 therapy (75, 76 and 79 year old).

**Table 2:** Post-surgery treatments received in a study cohort of 232 ≥70 y/o Caucasian female breast carcinoma patients stratified by ER/PR/HER2 subtype.

ER/PR/HER2 Subtype	Radiation Therapy		Hormonal Therapy		Chemotherapy	
*N* = 232	Received	Not Received	Received	Not Received	Received	Not Received
**Luminal A-like “Favorable” subtype**	33 (14%)	71 (30.6%)	59 (25.4%)	45 (19.3%)	13 (5.6%)	91 (39.2%)
**Luminal B /HER2− like “Favorable” subtype**	22 (9.4%)	52 (22.4%)	42 (18.1%)	32 (13.7%)	20 (8.6%)	54 (23.2%)
**Luminal B /HER2+ like Traditionally “Unfavorable” subtype**	6 (2.5%)	10 (4.3%)	10 (4.3%)	6 (2.5%)	5 (2.1%)	11 (4.7%)
**Nonluminal / HER2+ like Traditionally “Unfavorable” subtype**	3 (1.2%)	9 (3.8%)	1 (0.4%)	11 (4.7%)	4 (1.7%)	8 (3.4%)
**Triple negative-like “Unfavorable” subtype**	11 (4.7%)	15 (6.4%)	0 (0%)	26 (11.2%)	7 (3%)	19 (8.1%)
**Total (% of total)**	75 (32.3%)	157 (67.7%)	111/194 (57.2%)	83/194 (42.7%)	49 (21.1%)	183 (78.9%)

**Figure 1:**
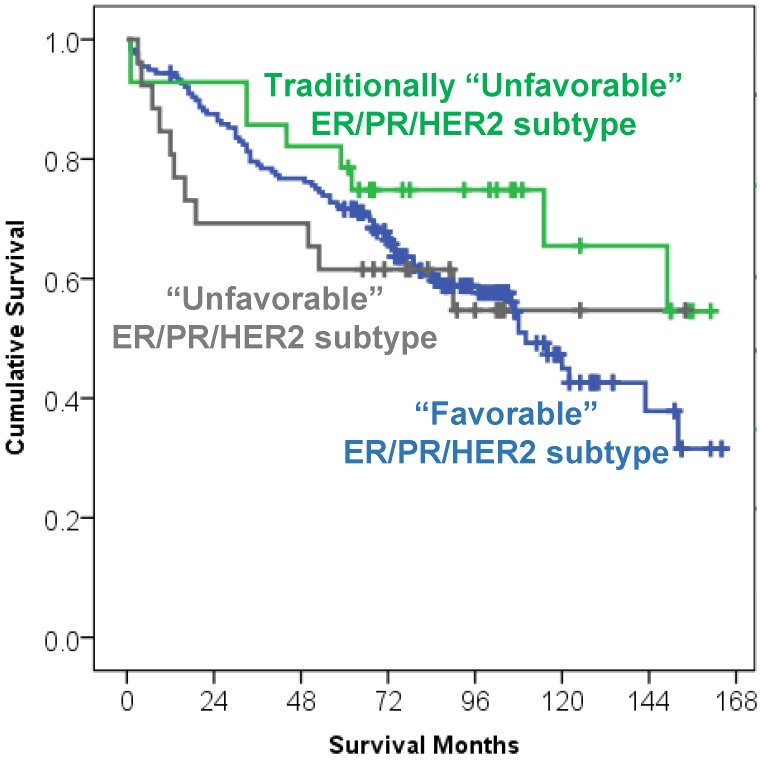
Kaplan Meier survival curve of 232 elderly breast carcinoma patients (≥70 y/o) stratified by the ER/PR/HER2 “favorable” (luminal A-like and luminal B/HER2− like), traditionally “unfavorable” (luminal B/HER2 positive like and non-luminal/HER2+ like) and “unfavorable” triple negative subtype. ER/PR/HER2 subtype had no significant impact on overall survival (*p* = 0.285).

Although a trend for better overall survival was noted in HER2+ breast carcinoma patients that were traditionally considered as an “unfavorable” breast carcinoma subtype [68% alive HER2+ patients at the end of the study period vs, 56% alive patients in ER+/PR+/HER2− (“favorable” subtype) or 58% alive patients in triple negative “unfavorable” subtype], the “favorable” breast carcinoma subtype was not significantly predictive of better overall survival (Figure 1, Kaplan Meier curve, *p* = 0.285).

**Figure 2:**
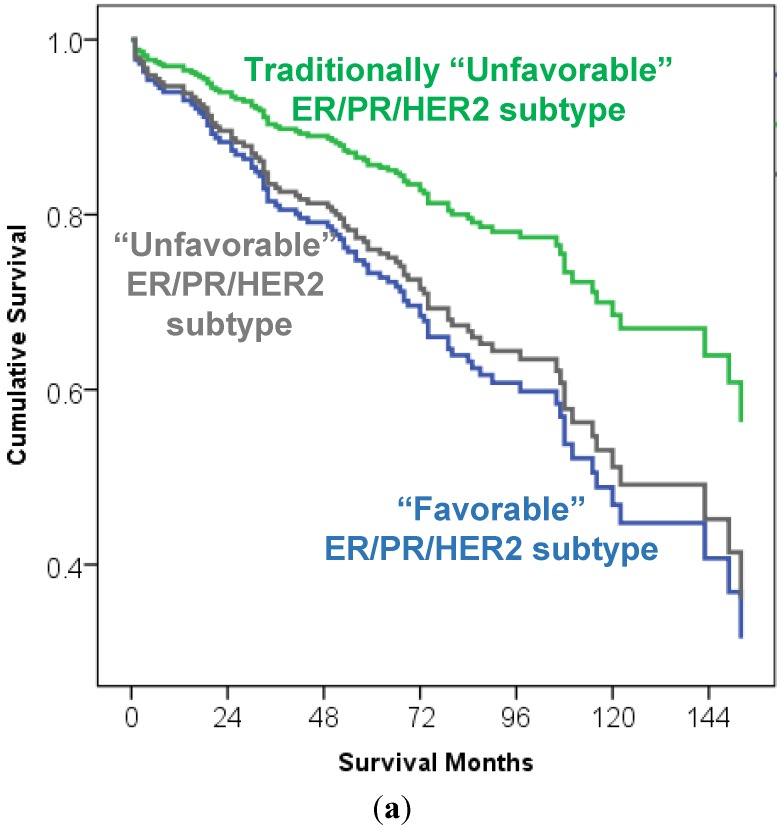

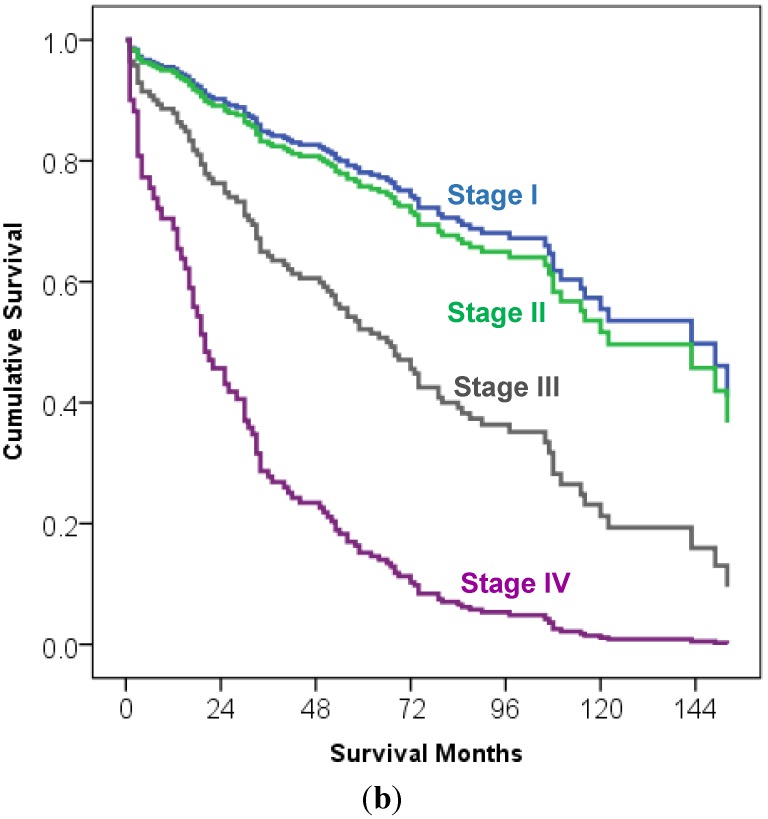
Overall survival curve of 232 elderly breast carcinoma patients (≥70 y/o) stratified by the ER/PR/HER2 subtypes [“favorable” (luminal A-like and luminal B/HER2− like), traditionally “unfavorable” (luminal B/HER2 positive like and non-luminal/HER2+ like) and “unfavorable” triple negative subtype], TNM stage and tumor grade (multivariate Cox regression analysis. (**a**) The ER/PR/HER2 subtype was not a significant predictor of overall survival (*p* = 0.095–0.95) (overall survival curve output by ER/PR/HER2 subtype); (**b**). TNM stage was a significant predictor of overall survival in advanced cancer stages (*p* < 0.001; stage III HR 2.74 95% CI 1.53–4.91; stage IV HR 7.28 95% CI 3.30–16.08), but there was no significant difference between TNM stage I and stage II in this analysis (*p* = 0.641) (overall survival curve output by TNM stage). The tumor grade was not a significant predictor of overall survival (*p* = 0.47) (curve not shown).

In Cox regression multivariate analysis, when ER/PR/HER2 subtypes were controlled for TNM stage and grade, a trend for better overall survival was again noted in HER2+ breast carcinoma patients that were traditionally considered as an “unfavorable” breast carcinoma subtype, but it did not reach statistical significance (*p* = 0.095; Figure 2a). There was no survival difference between TNM stage I and stage II, regardless of breast cancer subtype (*p* = 0.641). However, TNM stage was a significant predictor of overall survival in advanced stages: patients with stage III and stage IV breast carcinoma had significantly worse overall survival in comparison to patients with stage I and stage II breast carcinoma (*p* < 0.001; stage III HR 2.74 95% CI 1.53–4.91; stage IV HR 7.28 95% CI 3.30–16.08; Figure 2b). The tumor grade was not a significant predictor of overall survival (*p* = 0.47).

## 3. Discussion

We evaluated whether ER/PR/HER2 subtype and TNM stage of invasive breast carcinoma had a significant impact on overall survival in a cohort of 232 elderly Caucasian female patients (≥70 y/o) from our institution over a 10 year interval (January 1998–July 2008), with at least a subsequent 60 month follow-up period (median follow up 72 months).

We found that the majority of our elderly patients (76.8%) were of the “favorable” luminal A-like and luminal B/HER2 negative-like breast carcinoma subtype (ER+ and/or PR+, HER2−). When hormone receptor-positive HER2 positive patients (luminal B/HER2 positive patients) were added to ER+ and/or PR+, HER2− patients, a total of 83.6% of our patients had hormone receptor-positive breast carcinoma. The number of hormone receptor-positive patients in our study was in concordance with the percentage of elderly patients with “favorable” hormone receptor-positive breast carcinoma reported in the review article by O’Connor *et al.*, which reached up to 85% [11]. It was only slightly lower than the percentage published by Diab *et al.* [12] who analyzed tumor characteristics of breast carcinomas by age group in the San Antonio database and found that, on average, 89% of patients 65 years and older had hormone receptor-positive breast carcinomas. In Diab’s study [12], the percentage of HER2 positive patients was identical to that in our study (12%). A slightly lower percentage of patients in our study had triple negative-like breast carcinoma subtype (11.2%) in comparison to 15%–18% triple negative-like breast carcinoma subtype reported by Aapro and Wildiers [4] in their review of data estimating the number of triple negative breast carcinoma patients in the United States and Chinese breast cancer patients, respectively.

Other clinicopathologic characteristics of tumors in our patients, such as invasive ductal carcinoma being the most common histologic tumor type, tumor size most commonly below 2 cm, and approximately 19% of patients with 1–3 positive lymph nodes, were similar to the tumor characteristics reported in other studies [12,13]. The same was true for the grade of the tumor observed in our study (29.5% of grade 1, 45.6% of grade 2 and 24.5% of grade 3 tumors) when compared to the study on 41,390 women age 65 and older diagnosed with stage I to III breast carcinoma between 1991–1999 from the Surveillance, Epidemiology and End Results-Medicare linked database reported by Giordano *et al.* [13].

We observed a trend for better overall survival in HER2+ breast carcinoma patients that were traditionally considered as “unfavorable” breast carcinoma subtype over patients in “favorable” breast carcinoma subtype (ER and/or PR+, HER2−); however, it did not reach statistical significance. In fact, no ER/PR/HER2 subtype was significantly predictive of better overall survival. We are tempted to hypothesize that the observed trend may, perhaps, be a result of HER2+ patients receiving both anti-HER2 therapy and chemotherapy for treatment of HER2+ breast carcinoma but, due to the small number of these patients in our study population, this effect did not reach a statistical impact. Our future studies investigating the survival impact of variations in treatments that our patients received may answer this question, once we analyze a larger number of patients and have a longer follow-up period.

Nevertheless, results from this study demonstrate that ER/PR/HER2 status of breast carcinoma had no significant impact on overall survival in the elderly patients. The TNM stage was predictive of overall survival only in those that presented with stage III and stage IV breast cancer, whereas there was no survival difference between stage I and stage II cancers. These results are similar to our two previously published studies where ER/PR/HER2 status was not predictive of overall survival of Caucasian female breast carcinoma patients of all ages, irrespective of the classification system used, while TNM stage was predictive of overall survival primarily in advancing stages [9,10]. Possible causes for the results from our previous studies were attributed to the composition of our study population (we were only studying Caucasian female breast cancer patients), type of ER/PR/HER2 classification system used (St. Gallen breast carcinoma subtype classification or triple negative *vs.* non-triple negative breast carcinoma subtype), and the time period of the study (1998–2008) wherein screening, diagnostics, and treatment of breast carcinoma patients improved significantly over time periods that were observed before 1998. The decreased mortality observed in our “unfavorable” ER/PR/HER2 subtypes in our elderly patients is perhaps due to better personalized treatment which, in turn, resulted in a clinical loss of distinction between “favorable” and “unfavorable” ER/PR/HER2 breast carcinoma subtypes, as we mentioned earlier as a possibility for at least HER2+ patients. It is interesting to note that, in at least two other different studies, elderly patients with “unfavorable” triple negative breast carcinoma phenotype had a better, or the same, outcome when compared to their corresponding younger cohort [14,15]. This survival outcome occurred in spite of a significantly lower use of chemotherapy and radiotherapy in the elderly patients, raising the possibility that a different tumor biology exists between younger and older patients [4]. It is also possible that elderly patients with “favorable” subtypes are being undertreated, resulting in outcomes that paralleled the “unfavorable” subtypes. This possibility should be explored more since we observed that higher percentages of patients in our study with “unfavorable” subtypes received chemotherapy in comparison to patients with “favorable” ER+/PR+/HER2− subtype.

## 4. Materials and Methods

### 4.1. Selection and Description of Participants

The study was carried out following the approval from the Institutional Review Board of the University of Tennessee Medical Center at Knoxville (IRB project number: 3156; date of initial approval: 25 January 2011).

Our study was limited to Caucasian females only due to our institution’s geographic location in East Tennessee, where greater than 90% of the total patient population are Caucasian.

From our institution’s Cancer Institute prospectively monitored database, 1253 Caucasian female patients diagnosed with primary invasive breast carcinoma between 1 January 1998 and 1 July 2008 (10 year period) were identified. Of these, 232 women were 70 years or older (18.5% of population), had complete data and entered our study (median age 76 years, age range 70–98 years). Since HER2 testing of all breast carcinomas in our institution started in 1998, after FDA approved anti-HER2 therapy for metastatic breast carcinomas, this 10 year time frame allowed us to include patients with complete ER/PR/HER2 subtypes and have a minimum of five years of follow-up data, with 1 August 2013 being the last day of follow-up.

Invasive carcinoma histologic type was determined based on World Health Organization classification [16].

Two-hundred-thirty-two Caucasian women were eligible for the study and the following clinicopathologic characteristics were analyzed: histologic tumor type, tumor grade, tumor size, ER/PR/HER2 status, TNM stage, treatments received and overall survival status.

### 4.2. Determination of Clinicopathologic Tumor Characteristics and Breast Cancer Subtype

TNM (tumor-node-metastasis) stage was established based on the AJCC staging manual edition that was in place at the time of final pathologic evaluation [17,18] but was re-staged to the newest 7th edition of AJCC staging manual in our study [19]. Then, stage IA and IB were combined to stage I, stage IIA and IIB to stage II, and stage IIIA, IIIB and IIIC to stage III.

Immunohistochemical methods were used to determine ER, PR and HER2 status at the time of the patient’s cancer evaluation on a selected tumor block of the needle core biopsy tissue and/or a tumor block after final surgical excision. ER and PR was considered positive with more than 1% of the nuclear staining upon re-evaluation based on the newest ASCO/CAP guidelines for immunohistochemical testing for ER and PR in breast cancer [20]. A 3+ immunoreactivity HER2 score was considered as overexpressed for HER2, and 0+ or 1+ immunoreactivity was considered negative. Tumors with 2+ immunohistochemical scores were sent to an independent reference laboratory for fluorescence in-situ hybridization (FISH) until December 2007, and after December 2007, an independent reference laboratory performed HER2 FISH analysis for all tumors. A ratio of >2.2 of HER2-neu copies to CEP17 copies on chromosome 17 was considered positive for HER2-neu gene amplification [21].

Ki-67 proliferation index was determined in the area with highest Ki-67 nuclear labeling. From a total of 300 counted cells, proliferating and non-proliferating, the percentage of proliferating cells was calculated, and reported as percentage of proliferating cells.

**Figure 3:**
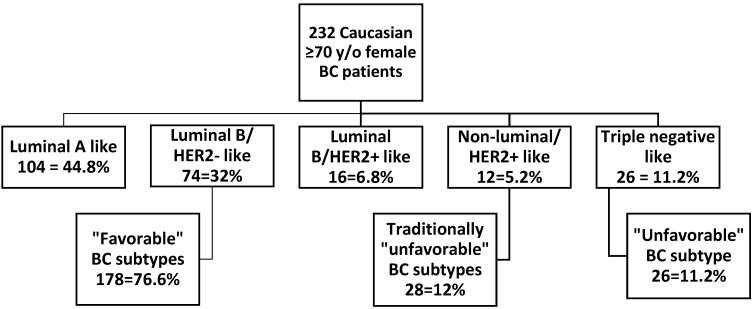
ER/PR/HER2 breast carcinoma subtypes in a study cohort of 232 ≥70 y/o Caucasian female breast carcinoma patients. Study patients are grouped based on the ER/PR/HER2 phenotype into “favorable” (luminal A-like and luminal B/HER2− like), traditionally “unfavorable” (luminal B/HER2 positive like and non-luminal/HER2+ like), and “unfavorable” triple negative subtype.

Patients were grouped according to their ER/PR/HER2 subtypes into five groups, per the 2011 St. Gallen International Expert Consensus recommendations [22] (Figure 3) as follows: group 1—Luminal A-like subtype = ER and/or PR positive, HER2 negative and Ki-67 proliferation index less than 14%; group 2—Luminal B/HER2 negative-like subtype = ER and/or PR positive, HER2 negative and Ki-67 proliferation index more than 14%; group 3—Luminal B/HER2 positive-like subtype = ER and/or PR positive, HER2 positive and any Ki-67 proliferation index; group 4—HER2 positive/non-luminal like subtype = ER and PR negative, HER2 positive and any Ki-67 proliferation index, and group 5—Triple negative subtype = ER and PR negative and HER2 negative and any Ki-67 proliferation index. These groups were further subclassified into “favorable” subtype = luminal A-like and luminal B/HER2 negative subtype and two “unfavorable” subtypes: (1) HER2+ subtype = luminal B/HER2 positive subtype and non-luminal HER2 positive subtype (“traditionally considered unfavorable”) and (2) “unfavorable” triple negative subtype.

### 4.3. Patient’s Inclusion into the Study, Treatment and Survival

Caucasian female patients 70 years and older, diagnosed with stage I to IV primary invasive breast carcinoma from 1 January 1998 to 1 July 2008 with complete ER/PR/HER2 data were included into our study. The patients were excluded if diagnosed with synchronous or metachronous malignancies of other organs.

The patients were treated with surgery, chemotherapy, and radiation therapy in accordance with the standard treatment protocol at the time of the diagnosis. This was based on NCCN guidelines or similar evidence-based data at the time of diagnosis and was individualized based on the patient’s comorbidities [23,24]. For patients receiving chemotherapy, the standard treatment method was to use a second-generation chemotherapy regimen for Stage I breast cancer and a third-generation regimen for Stage II and III. Anti-hormonal treatment was given to ER positive patients (aromatase inhibitors or tamoxifen). Anti-HER2 therapy was given to HER2 positive patients when diagnosed with systemic metastatic disease up to October 2005 and, after October 2005, eligible HER2+ patients received anti-HER2 therapy in addition to the above described chemotherapy regimens. Follow-up status and outcome information were obtained from our cancer registry database, with 1 August 2013 being the last date of follow-up for patients who were still alive.

### 4.4. Statistical Analysis

Clinicopathologic characteristics of breast carcinomas in our elderly patients were analyzed using frequency statistics. The Kaplan-Meier curve was used as a univariate analysis to establish how ER/PR/HER2 subtypes differed in terms of overall survival. The Cox Regression curve was used as a multivariate analysis to determine how ER/PR/HER2 subtypes differed in terms of survival when controlled for TNM stage and grade.

SPSS Version 19 (SPSS Inc., Chicago, IL, USA) was used for statistical analysis. Statistical significance was assumed at a *p* < 0.05 level.

## 5. Conclusions

Results from our study on elderly Caucasian female breast carcinoma patients from our institution demonstrated that ER/PR/HER2 status was not predictive of overall survival, but TNM stage was predictive of overall survival, and are similar to two of our previously published studies on Caucasian female breast cancer patients of all ages [9,10]. Since standardized treatment recommendations for patients >70 y/o are less strictly defined than for other age groups, further studies investigating treatments that elderly patients receive are warranted, with a goal to reconcile and stratify given therapy with outcome.
